# Interspecific Differences in Carbon and Nitrogen Metabolism and Leaf Epiphytic Bacteria among Three Submerged Macrophytes in Response to Elevated Ammonia Nitrogen Concentrations

**DOI:** 10.3390/plants13111427

**Published:** 2024-05-21

**Authors:** Heyun Wang, Kuang Chen, Hui Jin, Rui Hu

**Affiliations:** Key Laboratory of Intelligent Health Perception and Ecological Restoration of River and Lake, Ministry of Education, Innovation Demonstration Base of Ecological Environment Geotechnical and Ecological Restoration of Rivers and Lakes, School of Civil Engineering, Architecture and Environment, Hubei University of Technology, Wuhan 430068, China; sihenchen@gmail.com (K.C.);

**Keywords:** submerged macrophytes, NH_4_-N stress, leaf epiphytic bacteria, interspecific differences

## Abstract

Submerged macrophytes in eutrophic aquatic environments adapt to changes in ammonia nitrogen (NH_4_-N) levels by modifying their levels of free amino acids (FAAs) and soluble carbohydrates (SCs). As symbionts of submerged macrophytes, epiphytic bacteria have obvious host specificity. In the present study, the interspecific differences in the FAA and SC contents of *Hydrilla verticillata* (Linn. f.) Roylep, *Vallisneria natans* Hara and *Chara braunii* Gmelin and their leaf epiphytic bacterial communities were assessed in response to increased NH_4_-N concentrations. The results revealed that the response of the three submerged macrophytes to NH_4_-N stress involved the consumption of SCs and the production of FAAs. The NH_4_-N concentration had a greater impact on the variation in the FAA content, whereas the variation in the SC content was primarily influenced by the species. At the phylum level, the relative abundance of Nitrospirota on the leaves exhibited specific differences, with the order *H. verticillata* > *V. natans* > *C. braunii.* The dominant genera of epiphytic bacteria with denitrification effects on *V. natans, H. verticillata* and *C. braunii* leaves were *Halomonas, Acinetobacter* and *Bacillus*, respectively. When faced with NH_4_-N stress, the variation in epiphytic bacterial populations associated with ammonia oxidation and denitrification among submerged macrophytes could contribute to their divergent responses to heightened nitrogen levels.

## 1. Introduction

Intense rainfall events resulting from the effects of global climate change cause substantial fluctuations in the availability of nitrogen for plants in both terrestrial and aquatic ecosystems [1,2,3,4,5]. In aquatic environments, an increase in the ammonia nitrogen (NH_4_-N) concentration is a major factor contributing to the decline of underwater plants. Elevated NH_4_-N concentrations can lead to leaf wilting and yellowing and abnormal root growth [6], and hinder the development and growth of seeds and young plants [7,8]. Submerged plants experience the physiological inhibition of protein synthesis, leading to an imbalance in the carbon and nitrogen metabolism, ultimately inhibiting plant growth [9,10]. Numerous studies have indicated that NH_4_-N concentrations exceeding 1.0 mg L^−1^ have detrimental effects on submerged plants, and they counteract the toxicity of NH_4_-N by metabolizing NH_4_^+^ into free amino acids (FAAs) and organic amines through the internal consumption of soluble sugars (SCs). This process was elucidated by the findings of Cao et al. [11,12,13], Wang et al. [14] and Jin et al. [15]. Nevertheless, notable variations were observed among the responses of different species to increased levels of NH_4_-N [15,16,17,18,19].

Biofilms consist of intricate aggregates of bacteria that have a specific three-dimensional structure, are connected to solid surfaces [20] and are tightly encapsulated by extracellular polymeric substances (EPSs) secreted by the attached microorganisms, which create physical barriers for material transfer and form a “closed microenvironment” that can induce biological interactions [21], whereas ‘epiphytic biofilm’ occurs on the aboveground surfaces of macrophytes [22]. The relationship between epiphytic biofilms and plants involves intricate connections, such as competition for nutrients [23,24] and the collaborative breakdown of contaminants in the water [25,26]. The community structure of the epiphytic microorganisms of submerged plants is closely related to environmental variables, which can directly or indirectly affect attached microorganisms by inducing changes in submerged vegetation [27,28,29]. For example, a high NH_4_-N concentration could increase the microbial colony area and reduce the heterogeneity of the *Vallisneria natans* and *Hydrilla verticillata* leaf surface [30]. Increased NH_4_-N levels can promote the growth of attached microorganisms in *V. natans* and interfere with the microbial food web in epiphytic biofilms, which may contribute to the growth of epiphytic bacteria and algae [31]. Furthermore, an increase in total nitrogen significantly increases the abundance of ammonia oxidation genes (AMOA) and denitrification genes (nirK, nirS, napAa and dcnorB) in the biofilms of *V. natans*, *H. verticillata* and *Potamogeton malaianus* [32].

Leaf epiphytic bacteria on submerged macrophytes are affected by the growth environment [33,34,35,36,37] as well as the growth status of plants [38]. The characteristics of host plants, such as the leaf architecture/shape and chemical compounds [39,40,41,42,43], can affect epiphytic biofilms by providing a substrate for attachment [22], and epiphytic bacteria exhibit obvious host specificity [35,42,44]. Furthermore, interspecific differences in the plant physiological metabolism can affect the composition of foliar bacterial communities. For example, phenolic allelochemicals secreted by the submerged plants *Myriophyllum spicatum* and *H. verticillata* affect the composition of bacteria attached to their leaves [30,41,42]. The current investigation focused on the variations in FAAs and SCs among three submerged macrophyte species, *H. verticillata*, *V. natans* and *Chara braunii* Gmelin, which have different growth forms. The objective of this study was to examine the effects of elevated NH_4_-N concentrations on the variation in FAAs, SCs and leaf epiphytic bacterial communities. This was achieved using a microcosm experiment system, with the aim of uncovering the interspecific differences in the carbon and nitrogen metabolism and leaf epiphytic bacterial communities in response to increased NH_4_-N concentrations. We made the following assumptions: (1) there would be interspecific differences in the carbon and nitrogen metabolism of submerged macrophytes in response to increased ammonia concentrations; (2) different submerged macrophytes would exhibit differences in the dominant leaf epiphytic bacterial community correlated with ammonia oxidation and denitrification functions in response to increased NH_4_-N concentrations.

## 2. Materials and Methods

### 2.1. Precultivation of Three Submerged Macrophytes

Three submerged macrophyte species with different growth forms (as shown in Figure S1), including *H. verticillate* (erect plants), *V. natans* (rosette-form plants) and *C. braunii* (bottom-dwellers) [45,46], were collected from Liangzi Lake (30°05′ N–30°18′ N, 114°21′ E–114°39′ E), a meso-eutrophic lake located in the middle reaches of the Yangtze River, in March 2021. The three species were precultured in 300 L organic glass cylinders under the same adaptation conditions, with 120 µmol m^−2^ s^−1^ light intensity, a 12:12 h light–dark cycle and an air temperature of 20 °C. The sediment was also taken from Liangzi Lake, but underwent a series of treatment processes before being used. These processes included selecting and removing stones, fully mixing and sun exposure to remove surface moisture. After preculture for one month, all macrophytes exhibited good growth.

### 2.2. Experimental Design

The study was conducted between 15 April and 28 April 2021 in 45 cylindrical transparent plastic buckets of the same volume (H = 38 cm, d = 30 cm) in a greenhouse located at the College of Environment and Water Conservancy Engineering, Hubei University of Technology (30°29′12″ N, 114°18′17″ E).

In the present study, the impact of increased NH_4_-N concentrations on macrophyte FAA and SC contents, the leaf epiphytic bacterial community and interspecific variations were examined using five different NH_4_-N concentrations (0, 1, 5, 10 and 20 mg L^−1^) and three submerged macrophyte species (*H. verticillata*, *V. natans* and *C. braunii*). For each species, 3 plastic buckets (H = 38 cm, d = 30 cm) with 10 L of tap water (NH_4_-N not detected) were used for each NH_4_-N concentration (0, 1, 5, 10 and 20 mg L^−1^); three plastic bowls (H = 10 cm, d = 10 cm) were placed in each bucket, and the plastic bowls were covered with 8 cm of the same sediment with the precultivation. Before being transplanted, well-growing macrophytes of uniform size were selected and cleaned with ultrapure water. In all, three macrophytes (or thalli) with a length of 8 cm for each species were used for each bucket and nine macrophytes (or thalli) were used for each concentration of NH_4_-N. After a week of acclimation, the NH_4_Cl solution was added to the plastic buckets to achieve the specified concentrations.

During the whole experiment, no ventilation and water exchange were performed, and the dissolved oxygen (DO), pH and oxidation-reduction potential (ORP) of the water were measured every day, and the levels of NH_4_-N, nitrite nitrogen (NO_2_-N), nitrate nitrogen (NO_3_-N) and total nitrogen (TN) were measured every other day. After each sampling, NH_4_-N was supplemented based on the measured NH_4_-N concentration to the designated concentration. Fourteen days later, fresh macrophyte leaves were collected for the measurement of the FAA and SC contents, and their epiphytic bacteria were removed and subjected to high-throughput sequencing.

### 2.3. Measurements of Aquatic Parameters, FAAs and SCs

The aquatic parameters (DO, pH and ORP) were measured daily using a portable instrument (YSI, ProQuatro, Yellow Springs, OH, USA). According to the established protocols for analyzing water and wastewater, Nath reagent spectrophotometry was used to detect NH_4_-N, N-(1-naphthyl)-ethylenediamine spectrophotometry was used to measure NO_2_-N, UV spectrophotometry was used to measure NO_3_-N and potassium persulfate UV spectrophotometry was employed to measure TN [47]. According to Yemm and Cocking [48], the FAA contents of macrophyte tissues were determined by the ninhydrin method, while the anthrone-sulfuric acid colorimetric method [49] was employed to determine the SC contents.

### 2.4. Analysis of Epiphytic Bacteria

To eliminate the presence of sizeable particles clinging to the leaves of two vascular plants, namely, *H. verticillata* and *V. natans*, as well as the thallus of *C. braunii*, approximately 0.5 g of fresh leaves (thalli) was first washed delicately three times using ultrapure water. Afterward, the leaves were transferred into a polyethylene tube with a volume of 50 mL, which was filled with 40 mL of sodium pyrophosphate solution (0.1 mol L^−1^ Na_4_P_2_O_7_·10H_2_O). After ultrasonication for 3 min, the samples were subjected to shaking at 225 r/min for 30 min, followed by an additional 3 min of ultrasonication. Finally, the suspension with the separated epiphytic bacteria was passed through a 0.22 μm (Millipore, Burlington, MA, USA) filter and kept at −20 °C for DNA extraction [44].

DNA extraction and detection analysis were performed by NovoGene (Beijing, China) Bioinformatics Technology Co., Ltd. The CTAB/SDS method was utilized to extract the total genomic DNA from the samples. The region to be amplified was 16S V4, with the forward primer being 515F (5′-CTCYCACCMCCCCCCCATA-3′) and the reverse primer being 806R (5′-CCACTACNVCCCTWTCTAAT-3′). Phusion^®^ High-Fidelity PCR Master Mix with GC Buffer and high-fidelity PCR Master Mix (New England Biolabs, Ipswich, MA, USA) were used to amplify the 16S rRNA genes. Initially, the samples underwent denaturation at 98 °C for 1 min. Subsequently, the samples underwent annealing at 50 °C for a period of 30 s, followed by elongation at 72 °C for 30 s. Finally, the sample was maintained at 72 °C for 5 min. Equal amounts of 1× loading buffer (containing SYBR Green) were combined with PCR samples, and electrophoresis was performed on a 2% agarose gel to assess the results. The samples containing a vibrant central band ranging from 400 to 450 base pairs were combined in equal proportions and then purified using the GeneJET Gel Extraction Kit by Thermo Scientific. The Illumina TruSeq DNA PCR-Free Library Preparation Kit (Illumina, San Diego, CA, USA) was utilized to construct sequencing libraries, which were then evaluated on a Thermo Scientific Qubit@ 2.0 fluorometer and Agilent Bioanalyzer 2100 system. Finally, the library was sequenced using an Illumina NovaSeq platform, resulting in the generation of 250 bp paired-end reads.

The Trimmomatic tool was used to filter and improve the quality of the raw sequences, Paired-end reads was assigned to samples based on their unique barcode and truncated by cutting off the barcode and primer sequence, and then merged using FLASH (V1.2.7) [50]. Sequence analysis was performed using QIIME (V1.9.1) [51]. Sequences exhibiting 97% resemblance were allocated to identical operational taxonomic units (OTUs). The RDP classifier was applied to select and analyze the representative sequences for each OTU [52]. All these sequences were submitted to the NCBI GenBank under the following project accession number: PRJNA1014426.

### 2.5. Statistical Analysis

Before statistical analyses, the data were square-root or log-transformed to achieve a normal distribution and homogeneity of variance. The variation in aquatic environmental factors (pH, DO, ORP, NO_3_-N, NO_2_-N and TN) with time and the NH_4_-N concentration was analyzed with repeated measures analysis of variance. The effects of the NH_4_-N concentration and species, as well as their interactions, on the macrophytes’ FAA and SC contents and the FAA/SC ratio were analyzed using a two-way ANOVA. The differences between various treatments were analyzed using the t test. Using the ‘vegan’ [53] package in R, the relationships between the dominant bacterial groups on the leaf surface and the physical and chemical characteristics of the aquatic environment were analyzed through redundancy analysis (RDA). Additionally, the variation among the primary bacterial clusters on the leaf surfaces of the three macrophyte species was also assessed. The RDA results were plotted using the ‘ggplot2’ [54] package, and confidence ellipses were plotted using the ‘stat_ellipses’ function to show the differences in the primary bacterial clusters on the leaf surface of the three macrophyte species and their changes with the increase in the NH_4_-N concentration. Moreover, a correlation analysis between the abundances of genera correlated with ammonia oxidation and denitrification and the NH_4_-N concentration was performed using the ‘psych’ [55] package, and heatmaps were generated using the ‘pheatmap’ [56] package.

## 3. Results

### 3.1. Changes in Aquatic Environmental Factors

**Figure 1:**
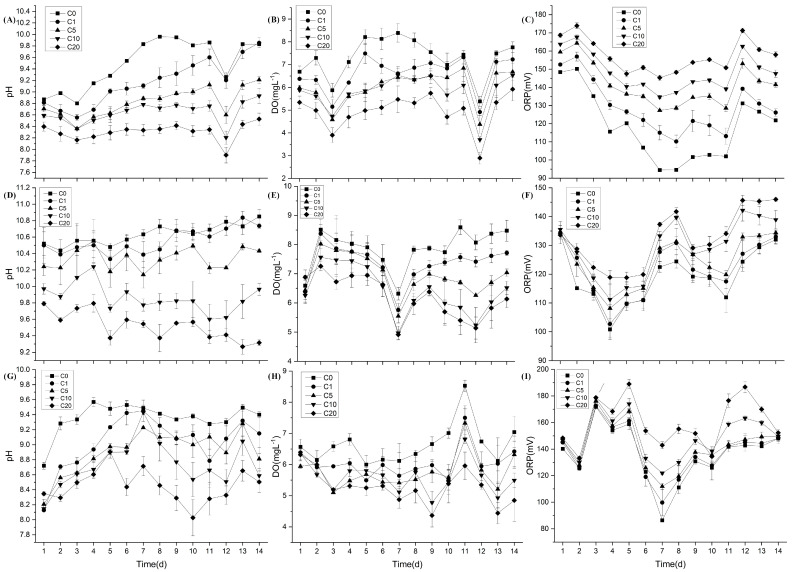
Variation in pH, dissolved oxygen (DO) and oxidation-reduction potential (ORP) in solutions containing *V. natans* (**A**–**C**), *H. verticillata* (**D**–**F**) and *C. braunii* (**G**–**I**) under different NH_4_-N concentrations (C0: 0 mg L^−1^, C1: 1 mg L^−1^, C5: 5 mg L^−1^, C10: 10 mg L^−1^ and C20: 20 mg L^−1^) (means ± SD, n = 3).

Repeated measures analysis of variance revealed that time and the NH_4_-N concentration significantly affected the pH, DO and ORP for the three macrophytes. The solution pH varied between 7.9 and 10.0 for *V. natans* (Figure 1A), between 9.3 and 10.8 for *H. verticillata* (Figure 1D) and between 9.0 and 10.4 for *C. braunii* (Figure 1G). The solution pH for *H. verticillata* was greater than that for the other two species. With an increasing NH_4_-N concentration, the solution pH significantly decreased (for *V. natans*, *p* < 0.001; for *H. verticillate, p* < 0.001; for *C. braunii*, *p* < 0.001). The solution DO concentration varied between 2.9 mg L^−1^ and 8.4 mg L^−1^ for *V. natans* (Figure 1B), between 4.9 mg L^−1^ and 8.6 mg L^−1^ for *H. verticillata* (Figure 1E) and between 4.4 mg L^−1^ and 8.5 mg L^−1^ for *C. braunii* (Figure 1H). With an increasing NH_4_-N concentration, the solution DO concentration significantly decreased (for *V. natans*, *p* < 0.001; for *H. verticillate*, *p* < 0.001; for *C. braunii*, *p* = 0.003). The solution oxidation-reduction potential (ORP) varied between 95 mV and 174 mV for *V. natans* (Figure 1C), between 101 mV and 146 mV for *H. verticillata* (Figure 1F) and between 86 mV and 189 mV for *C. braunii* (Figure 1I). With an increasing NH_4_-N concentration, the solution ORP significantly increased (for *V. natans*, *p* < 0.001; for *H. verticillate*, *p* = 0.007; for *C. braunii*, *p* < 0.001).

**Figure 2:**
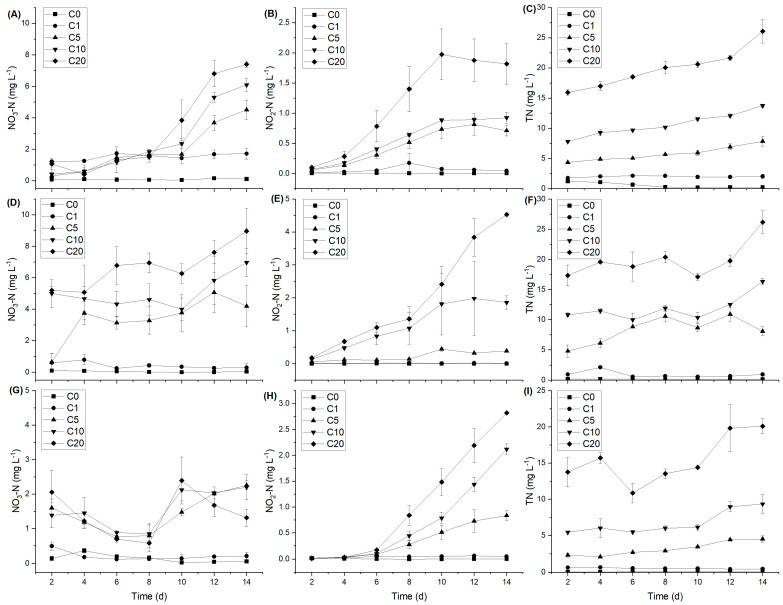
Variation in nitrate nitrogen (NO_3_-N), nitrite nitrogen (NO_2_-N) and total nitrogen (TN) in the solutions of *V. natans* (**A**–**C**), *H. verticillata* (**D**–**F**) and *C. braunii* (**G**–**I**) under different NH_4_-N concentrations (C0: 0 mg L^−1^, C1: 1 mg L^−1^, C5: 5 mg L^−1^, C10: 10 mg L^−1^ and C20: 20 mg L^−1^) (means ± SD, n = 3).

Repeated measures analysis of variance revealed that time and the NH_4_-N concentration significantly affected NO_3_-N, NO_2_-N and TN for the three macrophytes. The solution NO_2_-N concentration at two low NH_4_-N concentrations (0 and 1 mg L^−1^) was low but abruptly increased when the NH_4_-N concentration was above 5 mg L^−1^ for all three submerged species. A greater NO_2_-N concentration was detected in the *H. verticillata* solution compared to the solutions of the other two species (Figure 2). The variation in the concentration of solution TN was comparable to that of NO_2_-N.

### 3.2. Interspecific Differences between FAAs and SCs

By the end of the experiment, both *V. natans* and *H. verticillate* grew well at all NH_4_-N concentrations. However, *C. braunii* died at two high NH_4_-N concentrations of 10 mg L^−1^ and 20 mg L^−1^ on the 12th day of the experiment.

**Figure 3:**
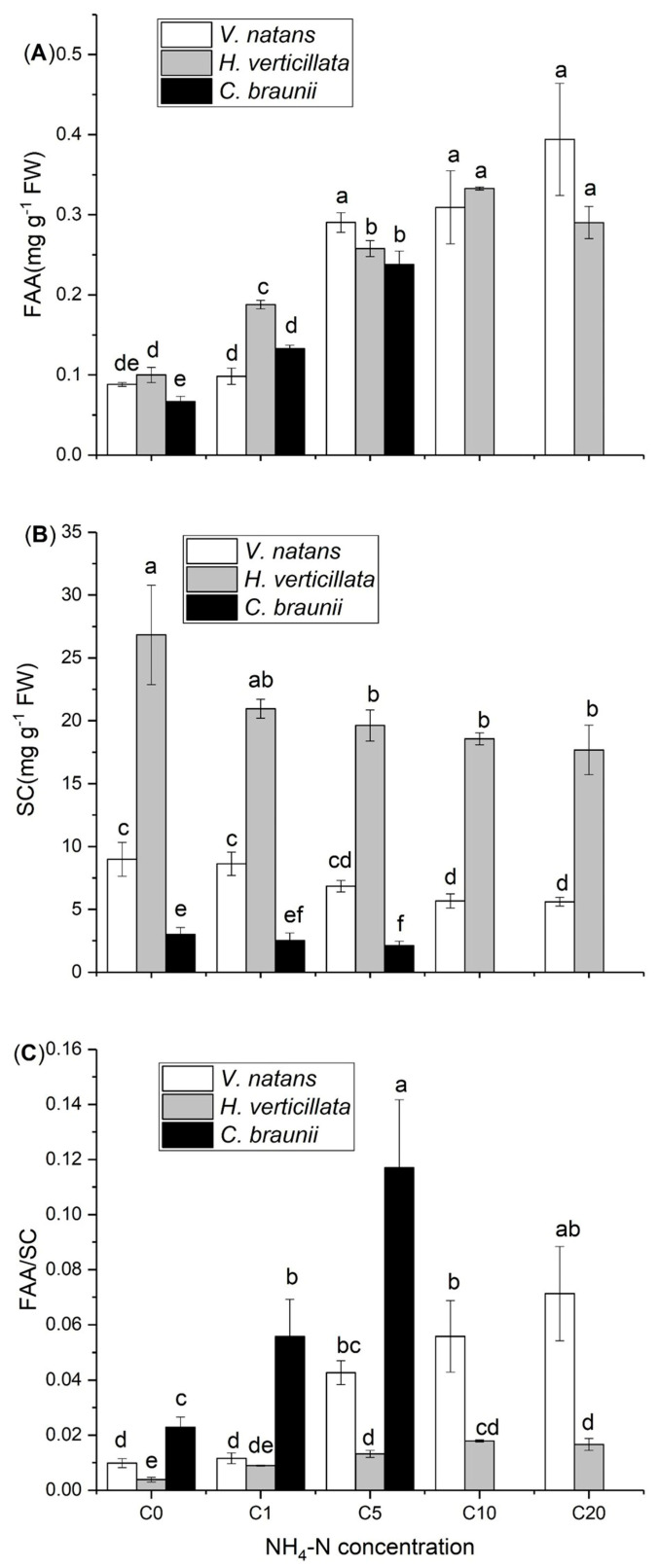
FAAs (**A**), SCs (**B**) and the FAA/SC ratio (**C**) of *V. natans*, *H. verticillata* and *C. braunii* under different NH_4_-N concentrations. Mean ± SD (n = 3). Significant differences between treatments are indicated by various letters (*p* < 0.05, *t* test).

Elevated NH_4_-N concentrations significantly increased the FAA content of the three submerged macrophyte species. However, the variation in the FAA content in response to the increase in the NH_4_-N concentration showed specific differences. When the NH_4_-N concentration was 1 mg L^−1^, the FAA content of *H. verticillata* was greater than that of the other two species. For both *V. natans* and *C. braunii,* the FAA content reached the highest level at 5 mg L^−1^ NH_4_-N, while it increased at 10 mg L^−1^ for *H. verticillate* (Figure 3A). At two elevated NH_4_-N concentrations (10 mg L^−1^ and 20 mg L^−1^), *C. braunii* died. Elevated NH_4_-N concentrations significantly decreased the SC contents of *V. natans* (at a concentration of 10 mg L^−1^) and *H. verticillata* (at a concentration of 5 mg L^−1^). The SC content at the three NH_4_-N concentrations (0, 1 and 5 mg L^−1^) decreased in the order of *H. verticillata > V. natans* > *C. braunii* (Figure 3B). Accordingly, the FAA/SC ratio of the three species significantly increased when the NH_4_-N concentration increased to 5 mg L^−1^, and the FAA/SC ratio at the three NH_4_-N concentrations (0, 1 and 5 mg L^−1^) decreased in the order *H. verticillata< V. natans* < *C. braunii* (Figure 3C). The two-way ANOVA indicated that the concentration of NH_4_-N had a greater impact on the variation in the FAA content, whereas the variation in the SC content was primarily influenced by the species (Table 1).

### 3.3. Correlations between Various Physical and Chemical Factors in Water and Leaf Epiphytic Bacterial Phyla

**Figure 4:**
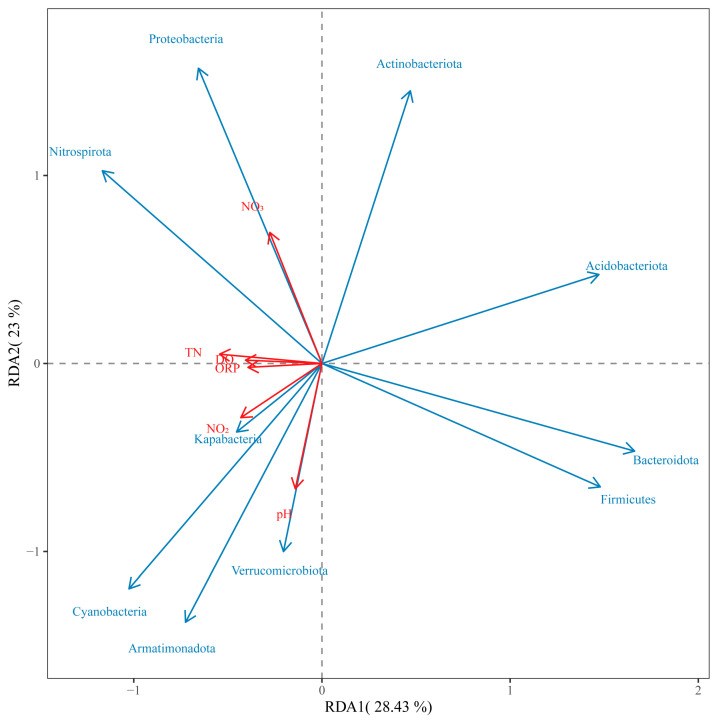
RDA of water environmental factors (pH, DO, ORP, NO_2_-N, NO_3_-N and TN) with leaf epiphytic bacterial phyla. The data on water environmental factors from the last measurement and bacterial phyla with the nine most abundant phyla, as well as Nitrospirota, were used for the analysis.

To examine the relationships between different physical and chemical factors affecting leaf epiphytic bacterial phyla with high relative abundances, RDA was performed. The results revealed that DO, ORP, NO_3_-N and TN were positively correlated with the abundances of Proteobacteria and Nitrospirota but negatively correlated with the abundances of Bacteroidota and Firmicutes. NO_2_-N and the pH were positively correlated with the abundances of Cyanobacteria, Armatimonadota, Verrucomicrobiota and Kapabacteria but negatively correlated with the abundances of Actinobacteriota and Acidobacteriota (Figure 4).

### 3.4. Interspecific Differences in the Response of Leaf Epiphytic Bacteria to Elevated NH_4_-N Concentrations

**Figure 5:**
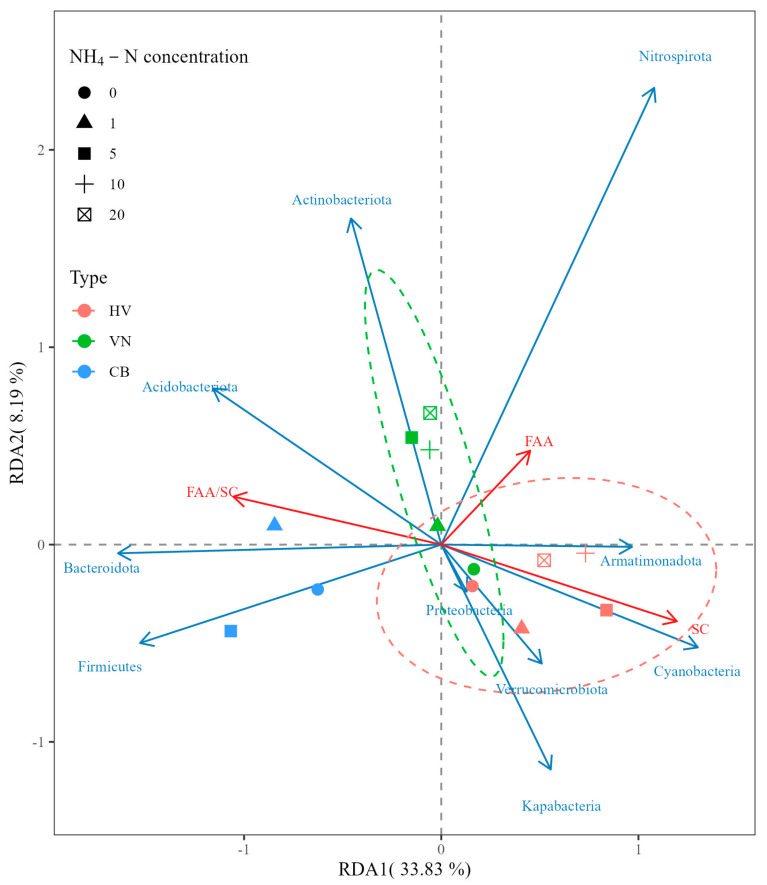
The interspecific differences in leaf epiphytic bacterial communities on *V. natans* (VN), *H. verticillata* (HV) and *C. braunii* (CB) with increasing NH_4_-N levels based on the RDA of physiological properties (FAAs, SCs and the FAA/SC ratio) and bacterial phyla with the greatest abundance, as well as Nitrospirota (different point shapes represent the experimentally controlled NH_4_-N concentration). The green ellipse means dominant epiphytic bacteria for *Vallisneria natans* (VN) while the red ellipse means dominant epiphytic bacteria for *Hydrilla verticillate* (HV).

**Figure 6:**
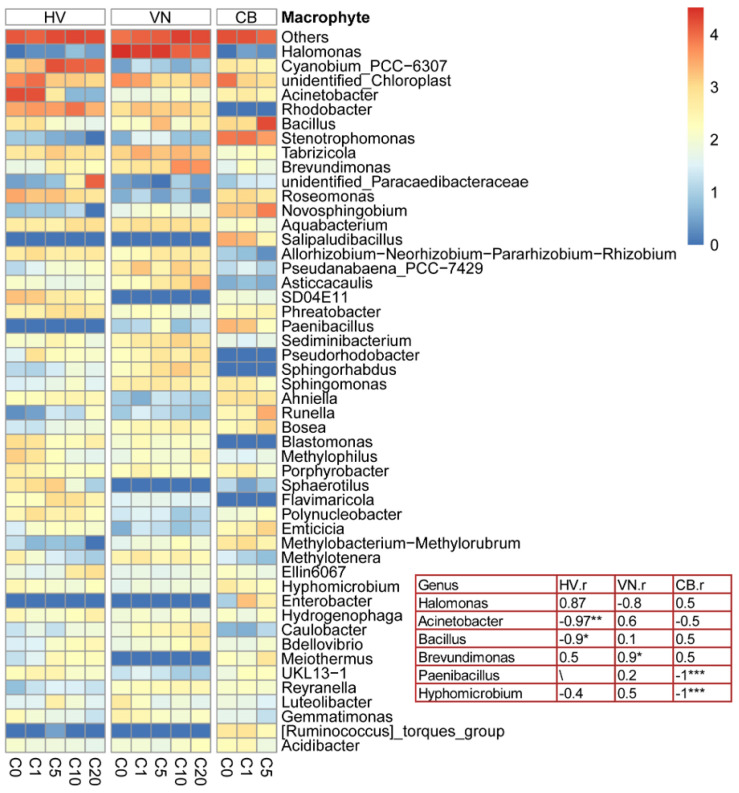
The top 50 genera based on the comprehensive abundance of *V. natans* (VN), *H. verticillata* (HV) and *C. braunii* (CB, no data shown for the C10 and C20 concentrations—the species died) and the abundance variation in the genera correlated with ammonia oxidation and denitrification function with NH_4_-N concentrations; log10 (x + 1) was used to construct a heatmap, where C0, C1, C5, C10 and C20 represent the NH_4_-N concentrations of 0, 1, 5, 10 and 20 mg L^−1^, respectively. Spearman’s correlation coefficients between genus abundance and the NH_4_-N concentration are shown in the table. “*” indicates a significant correlation (* *p* < 0.05, ** *p* < 0.01, *** *p* < 0.001). The selected genera are related to the nitrogen metabolism and their comprehensive abundance belong to the top 50.

RDA was applied to analyze the interspecific differences in leaf epiphytic bacterial communities with increasing NH_4_-N concentrations. As shown in Figure 5, the sample points of the same macrophytes under different NH_4_-N concentrations were relatively concentrated. When the NH_4_-N concentration was 0 mg L^−1^, the *H. verticillata* and *V. natans* sampling points were in close proximity, suggesting a similarity in the composition of the dominant bacterial phylum. However, as the NH_4_-N concentration increased, the separation of sampling points indicated a difference in the composition of the dominant bacterial phylum between the two species. The prevalence of Bacteroidetes and Firmicutes in the epiphytic bacteria of *C. braunii* exceeded that in the other two plants. At high concentrations of NH_4_-N (>1 mg L^−1^), the abundance of Actinobacteria among the epiphytic bacteria of *V. natans* was considerably greater than that in the other two macrophytes. The abundances of Cyanobacteria, Verrucomicrobia, Armatimonadota and Kapabacteria in the epiphytic bacteria of *H. verticillata* were considerably greater than those in the other two macrophytes. At the genus level, the abundances of *Bacillus*, *Stenotrophomonas*, *Novosphingobium* and *Salipaludibacillus* among the epiphytic bacteria of *C. braunii* were greater than those of the other two plants, while the abundance of *Halomonas* among the epiphytic bacteria of *V. natans* exceeded that of the other two macrophytes (Figure 6). At low concentrations of NH_4_-N (≤1 mg L^−1^), the abundance of *Acinetobacter* in the epiphytic bacteria of *H. verticillata* was considerably greater than that in the epiphytic bacteria of the other two macrophytes. As the NH_4_-N concentration increased, the abundances of *Cyanobium*_PCC-6307 and *Rhodobacter* in the epiphytic bacteria of *H. verticillata* were considerably greater than those in the other two macrophytes (Figure 6). At the genus level, for *V. natans, Brevundimonas* was positively correlated with the N levels, while, for *C. braunii*, *Paenibacillus* and *Hyphomicrobium* were negatively correlated with the N levels (Figure 6).

## 4. Discussion

### 4.1. Interspecific Differences in the FAA and SC Contents in Submerged Macrophytes in Response to Elevated NH_4_-N Concentrations

In some field surveys and controlled experimental studies, high concentrations of NH_4_-N have been shown to increase the FAA content and reduce the SC content in submerged macrophytes (such as *Ceratophyllum demersum*, *Myriophyllum spicatum* and *Potamogeton crispus*) [12,13,14,15,16,17,18,19,57,58,59]. In this experiment, the addition of 1.0 mg L^−1^ NH_4_-N significantly increased the FAA content in *H. verticillata* and *C. braunii*. Similar results have been found in *P. crispus* [11,13,15] and *C. demersum* [15]. However, the FAA and SC contents of identical species might vary due to differences in growth conditions. In the case of *V. natans,* the FAA and SC contents were measured to be 6 mg g^−1^ DW and 15 mg g^−1^ DW, respectively, according to Cao et al. [12]. At the 1 mg L^−1^ NH_4_-N level, the FAA and SC contents of *V. natans* were 0.09 mg g^−1^ FW and 8.63 mg g^−1^ FW, respectively (Figure 3A,B). In the present study, the variation in the FAA content in response to the increase in the NH_4_-N concentration exhibited specific differences. The SC content at three NH_4_-N concentrations (0, 1 and 5 mg L^−1^) decreased in the order *H. verticillata* > *V. natans* > *C. braunii* (Figure 3B). This aligned with our initial assumption. According to Cao et al. [11,12], FAA synthesis can also reduce the accumulation of NH_4_-N at the expense of SC. Therefore, a low content of SC in *C. braunii* could show that this species cannot withstand higher concentrations of NH_4_-N. This could explain why *C. braunii* died when the concentration of NH_4_-N was greater than 10 mg L^−1^. In fact, in recent decades, charophytes in shallow lakes have been declining and replaced by angiosperms due to the eutrophication of water bodies worldwide [60,61]. Therefore, we speculated that the toxicity of the increased NH_4_-N concentration caused by eutrophication to charophytes may also be one of the reasons for its decline. Furthermore, Yuan et al. noted that the specific difference could be ascribed to plant traits and their connectivity [5]. This suggests that morphological differences in *V. natans* and *H. verticillate*, as shown in Supplementary Figure S1, could exert a certain influence on the responses to an increased NH_4_-N concentration by affecting plant functional traits.

### 4.2. Interspecific Differences in the Response of Leaf Epiphytic Bacteria to Elevated NH_4_-N Concentrations

Depending on the growth environment and macrophyte species, Proteobacteria, Bacteroidetes, Actinobacteria and Firmicutes are the main types of bacteria on the leaves of submerged macrophytes [21,35,36,42,62,63]. In our study, Proteobacteria and Cyanobacteria were the dominant phyla among the leaf epiphytic bacteria of both *V. natans* and *H. verticillate.* Similar results were found for *V. natans* in a eutrophic environment [44,64] and for *Potamogeton cripus* and *Elodea canadensis* in a controlled microcosm experiment system with an exogenous phosphorus addition [65]. However, Proteobacteria and Firmicutes had the highest relative abundances among the leaf epiphytic bacteria of *C. braunii* (Supplementary Figure S1). A possible reason is that the genus *Bacillus* belongs to the phylum Firmicutes [66], and the relative abundance of *Bacillus* in the leaf epiphytic bacteria of *C. braunii* was much greater than that in the other two plants (Figure 6). Furthermore, the differences in the epiphytic bacteria between *C. braunii* and the other two could be ascribed to the differences in the surface properties, because calcium carbonate crystallization induced by the photosynthesis of *C. braunii* definitely created different surface properties. Wolters et al. [43] pointed out that the content of compounds such as calcium carbonate crystals affects the diversity and composition of leaf epiphytic bacterial communities.

However, the attributes of the epiphytic structure and function are associated with various environmental factors such as nitrogen nutrient availability [30,31,32]. In this study, RDA analysis revealed a strong association between the abundance of epiphytic-dominant bacteria and the nitrogen nutrient concentration (Figure 4). Furthermore, the α diversity of epiphytic bacteria varied with the change in the NH_4_-N concentration, and a higher α diversity was found at different NH_4_-N concentrations (20 mg L^−1^ for *V. natans*, 10 mg L^−1^ for *H. verticillata* and 1.0 mg L^−1^ for *C. braunii*; Supplementary Table S1). This confirmed that nitrogen nutrient variation could affect the epiphytic bacterial community. Similar results have been reported for the submerged plants *V. natans, H. verticillata* and *P. malaianus* under elevated NH_4_-N concentrations [31,32]. Furthermore, greater amounts of NO_2_-N and TN were detected under higher NH_4_-N concentrations (Figure 2). Simultaneously, there was an increase in the prevalence of Nitrospirota, a microorganism encoding the pathways for both ammonia and nitrite oxidation [67], as the levels of NO_3_-N and TN in the water increased (Figure 4). This may indicate that the accumulation of NH_4_-N and NO_2_-N in water induces the proliferation of Nitrospirota on macrophyte leaves. These results were in line with Yan et al. [32]’s findings that the increase in total nitrogen levels led to a notable increase in the presence of AMOAs within the biofilms of *V. natans*, *H. verticillata* and *P. malaianus*.

As mentioned above, epiphytic bacteria have obvious host specificity [35,42,44]. Based on the total abundances of three species, Nitrospirota (Comammox) was positively correlated with NO_3_-N or TN in the water (Figure 4), which significantly increased with the addition of NH_4_-N (Figure 2C,F,I). However, the relative abundance of Nitrospirota significantly differed with the same amount of NH_4_-N added on the leaves of different submerged macrophytes, exhibiting the order *H. verticillata* > *V. natans* > *C. braunii* (Supplementary Figure S1). Since Nitrospirota encodes pathways for both ammonia and nitrite oxidation [67], and has a high affinity for ammonia [68], the relative abundance of Nitrospirota on the leaf surface of submerged macrophytes indicates the ability of the system composed of macrophytes and attached bacteria to remove NH_4_-N. Moreover, these findings may also indicate the adaptability of the macrophyte species to ammonia nitrogen. Our results showed that, when the concentration of NH_4_-N was greater than 10 mg L^−1^, *C. braunii* macrophytes died, which was consistent with the lower abundance of Nitrospirota on charophyte leaves (Supplementary Figure S1). According to Behrendt et al. [69], *Paenibacillus* exhibits ammonia oxidation activity. The abundance of the genus with high comprehensive abundance significantly decreased with an increasing NH_4_-N concentration (Figure 6), which could explain the intolerance of *C. braunii* to high NH_4_-N concentrations. The current investigation also revealed that the abundances of Bacteroidetes and Firmicutes in the epiphytic bacteria of *C. braunii* was notably greater than that in the other two plants across three distinct NH_4_-N concentrations (0, 1 and 5 mg L^−1^) (Figure 5). At high concentrations of NH_4_-N (>1 mg L^−1^), the abundance of Actinobacteria in the epiphytic bacterial community of *V. natans* was considerably greater than that in the epiphytic bacterial communities of *H. verticillata* and *C. braunii*. These findings revealed distinct variations in epiphytic bacteria among the submerged macrophytes. As the provision of the substrate for attachment, host plant characteristics such as the life form and leaf architecture/shape [39,42] could affect the epiphytic biofilm. We speculate that the differences in the growth form and leaf structure (as shown in Supplementary Figure S1) may also be one of the reasons for the distinct variations in epiphytic bacteria among the submerged macrophytes. Furthermore, considering cleaning macrophytes with ultrapure water does not necessarily eliminate the difference in leaf epiphytic bacteria communities before acclimatization treatments, and distinct variations in epiphytic bacteria may also be due to the differences in the initial material.

Submerged plants and their epiphytic bacteria have synergistic effects on the purification of pollutants in lakes [70]. According to Pang et al. [63], *Halomonas*, *Acinetobacter*, *Bacillus* and *Rhodobacter* all have denitrification functions, and their abundances on the leaves of three plants of different life forms exhibited specific differences. In the present study, the dominant genera of epiphytic bacteria on the leaves of *V. natans, H. verticillata* and *C. braunii* were *Halomonas, Acinetobacter* and *Bacillus*, respectively. Furthermore, the abundance of *Rhodobacter* (3.92–13.65%) in *H. verticillata* was greater than that in *V. natans* (0.6–3.3%) and *C. braunii* (Figure 6). Furthermore, the abundance of *Hyphomicrobium,* a denitrifier [71], decreased significantly with an increasing ammonia nitrogen concentration, indicating a decrease in the nitrogen removal capacity. These findings revealed that distinct variations in epiphytic bacteria were correlated with denitrification among submerged macrophytes. This supported our second assumption. This could explain partly the differences in N removal among the three submerged macrophyte species. Similar results were reported by Yan et al. [31]. It was well known that sediment bacteria communities are involved the removal of N in water, although the abundance of denitrifying bacteria in the sediment was found to be lower than that in the epiphytic biofilm [63]. In our study, it is very difficult to deny the potential influence of sediment bacterial communities in N removal, since the sediment only had the same pretreatment but with no disinfection.

Hence, when faced with NH_4_-N stress, the variation in epiphytic bacterial populations among submerged macrophytes could contribute to the divergent responses of submerged species to heightened nutrient levels. When the sediment is the same, the differences in the symbiotic relationships between the submerged macrophytes and their epiphytic bacteria will eventually lead to differences in the survival and competitive advantages of submerged macrophytes in eutrophic lakes.

## Figures and Tables

**Table 1:** The explained variance based on the two-way ANOVA of species and NH_4_-N levels for FAA, SC and the FAA/SC ratio.

Parameters	Species (S)	NH_4_-N (N)	S × N	Error
FAA	0.47 ns	85.71 ***	7.96 ***	5.85
SC	90.58 ***	3.87 ***	2.82 **	2.74
FAA/SC	42.55 ***	31.91 ***	17.02 ***	8.51

The percentages are indicated by numbers (ns means *p* > 0.05, ** *p* < 0.01, *** *p* < 0.001).

## Data Availability

Data are available on request to the authors.

## Supplementary Materials

The following supporting information can be downloaded at: https://www.mdpi.com/article/10.3390/plants13111427/s1, Figure S1: Experimental facilities, experimental design and selected macrophytes; Figure S2: The top nine abundant phylum-level bacteria, as well as Nitrospirota, on the leaves of *V. natans*, *H. verticillate* and *C. braunii,* under different NH_4_-N concentrations; Table S1: The α diversity index of epiphytic bacteria on the leaves of *V. natans*, *H. verticillate* and *C. braunii* under different NH_4_-N concentrations.
