# Supplementary material for: Interspecific Differences in Carbon and Nitrogen Metabolism and Leaf Epiphytic Bacteria among Three Submerged Macrophytes in Response to Elevated Ammonia Nitrogen Concentrations
Source: Plants (Basel). 2024 May 21;13(11):1427. doi: 10.3390/plants13111427 (PMC11174776; doi:10.3390/plants13111427)
Supplement: Supplementary file 1 [file plants-13-01427-s001.zip › supplementary materials/Supplementary Figure 1.docx]

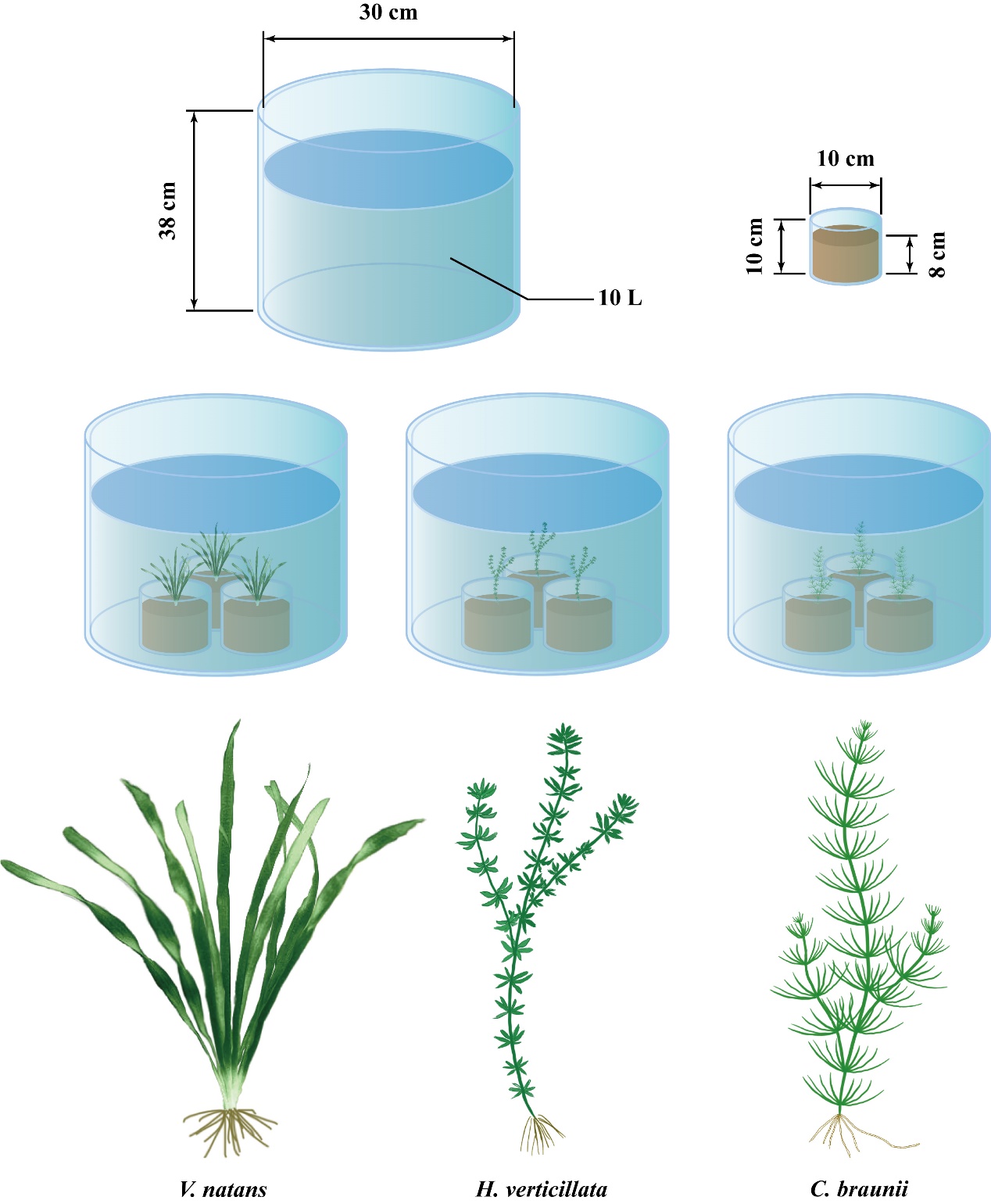


Supplementary Figure 1 Experimental facilities、experimental design and selected macrophytes.
