# Supplementary material for: Interspecific Differences in Carbon and Nitrogen Metabolism and Leaf Epiphytic Bacteria among Three Submerged Macrophytes in Response to Elevated Ammonia Nitrogen Concentrations
Source: Plants (Basel). 2024 May 21;13(11):1427. doi: 10.3390/plants13111427 (PMC11174776; doi:10.3390/plants13111427)
Supplement: Supplementary file 1 [file plants-13-01427-s001.zip › supplementary materials/Supplementary Figure 2.docx]

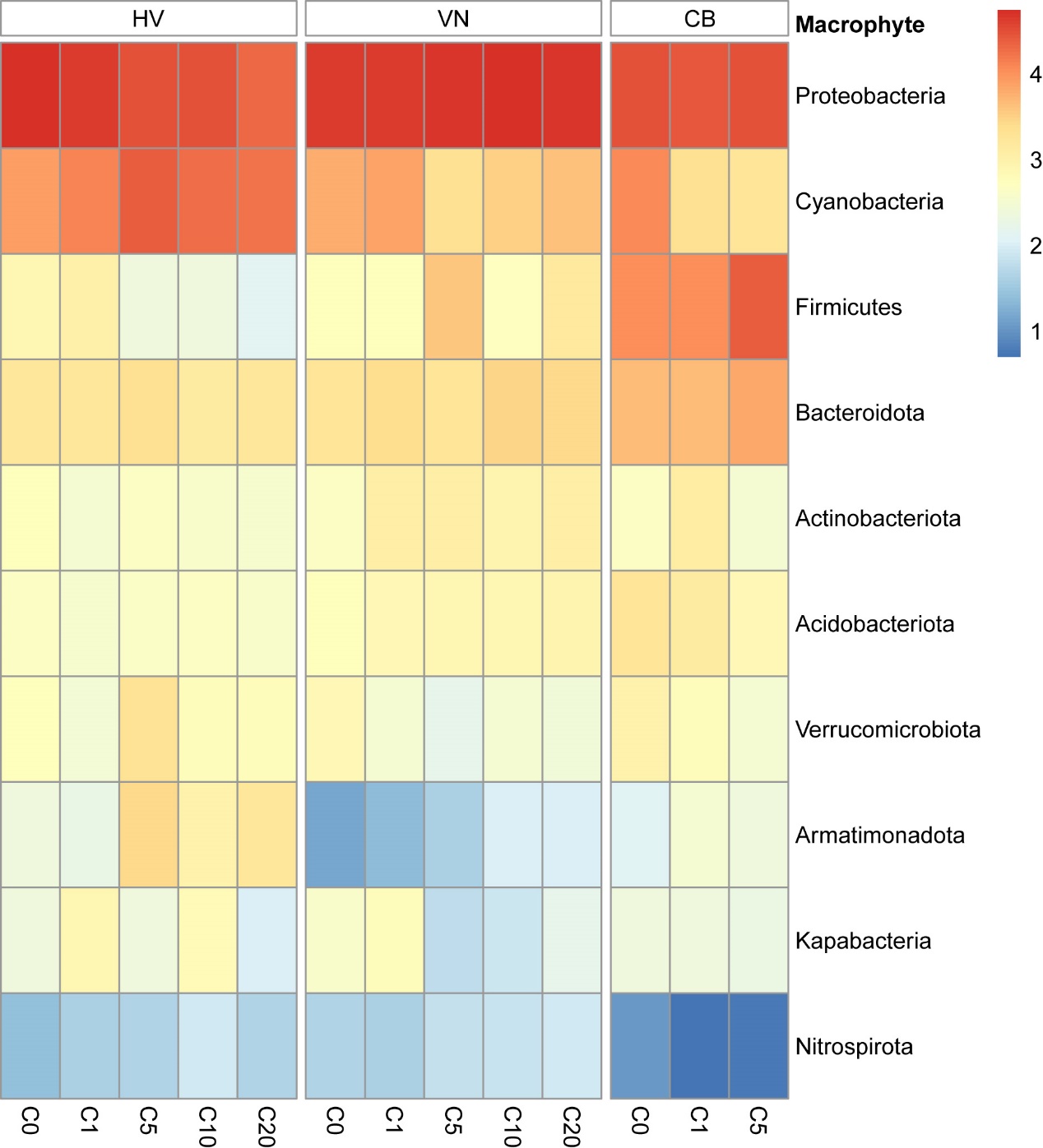


Supplementary Figure 2 The top nine abundant phylum-level bacteria, as well as Nitrospirota, on the leaves of *V. natans* (VN), *H. verticillate* (HV) and *C. braunii* (CB)*,* under different NH_4_-N concentration (The color depth represented the log10 of the relative abundance of selected bacteria, C0: 0 mg L^-1^, C1: 1 mg L^-1^, C5: 5 mg L^-1^, C10: 10 mg L^-1^, C20: 20 mg L^-1^).
