# Supplementary material for: Interspecific Differences in Carbon and Nitrogen Metabolism and Leaf Epiphytic Bacteria among Three Submerged Macrophytes in Response to Elevated Ammonia Nitrogen Concentrations
Source: Plants (Basel). 2024 May 21;13(11):1427. doi: 10.3390/plants13111427 (PMC11174776; doi:10.3390/plants13111427)
Supplement: Supplementary file 1 [file plants-13-01427-s001.zip › supplementary materials/Supplementary table 1.docx]

Supplementary Table 1 α Diversity index of epiphytic bacteria on the leaves of *V. natans*, *H. verticillate* and *C. braunii* under different NH_4_-N concentrations

| Species | NH_4_-N (mg L^-1^) | Shannon | Simpson | Chao1 | ACE |
| --- | --- | --- | --- | --- | --- |
| *V. natans* | 0 | 4.74 | 0.716 | 1571.57 | 1532.49 |
|  | 1 | 5.89 | 0.853 | 1698.00 | 1718.16 |
|  | 5 | 5.56 | 0.834 | 1693.17 | 1720.29 |
|  | 10 | 6.12 | 0.933 | 1615.15 | 1632.40 |
|  | 20 | 6.53 | 0.939 | 1666.30 | 1689.14 |
| *H. verticillata* | 0 | 5.96 | 0.905 | 1602.85 | 1637.03 |
|  | 1 | 5.88 | 0.913 | 1529.50 | 1570.50 |
|  | 5 | 6.93 | 0.956 | 1947.53 | 1992.59 |
|  | 10 | 7.07 | 0.955 | 2213.50 | 2258.01 |
|  | 20 | 6.75 | 0.936 | 1913.07 | 1975.77 |
| *C. braunii* | 0 | 7.08 | 0.957 | 2132.38 | 2158.65 |
|  | 1 | 7.64 | 0.971 | 2248.83 | 2301.72 |
|  | 5 | 5.47 | 0.881 | 1643.96 | 1688.85 |
